# Racial, gender, sexual, and disability identities of the *Journal of the Medical Library Association*'s editorial board, reviewers, and authors

**DOI:** 10.5195/jmla.2021.1216

**Published:** 2021-04-01

**Authors:** Katherine G. Akers, JJ Pionke, Ellen M. Aaronson, Thane Chambers, John W. Cyrus, Erin R.B. Eldermire, Melanie J. Norton

**Affiliations:** 1 JMLA@journals.pitt.edu, Editor-in-Chief, *Journal of the Medical Library Association*; 2 pionke@illinois.edu, University Library, University of Illinois at Urbana-Champaign, Urbana, IL; 3 aaronson.ellen@mayo.edu, Mayo Clinic Libraries, Mayo Clinic, Rochester, MN; 4 thane@ualberta.ca, Cameron Library, University of Alberta, Edmonton, AB, Canada; 5 cyrusjw@vcu.edu, Tompkins-McCaw Library for the Health Sciences, Virginia Commonwealth University, Richmond, VA; 6 erb29@cornell.edu, Flower-Sprecher Veterinary Library, Cornell University, Ithaca, NY; 7 melanie.norton@yale.edu, Cushing/Whitney Medical Library, Yale University, New Haven, CT

## Abstract

The *Journal of the Medical Library Association (JMLA)* recently issued a call for submissions that recognize and address social injustices; speak to diversity, equity, and inclusion in our workforce and among our user populations; and share critical perspectives on health sciences librarianship as well as those on any topic within *JMLA*'s scope written by authors who are Black, Indigenous, or People of Color. We also committed to creating more equitable opportunities for authors, reviewers, and editorial board members from marginalized groups. As part of this effort, we conducted a demographic survey of all individuals who served as a member of the *JMLA* editorial board or reviewer or had submitted a manuscript to *JMLA* between 2018 and 2020. We found that most survey respondents are white, heterosexual, women and do not identify with a disability, meaning that *JMLA* is missing out on a diversity of perspectives and life experiences that could improve the journal's processes and policies, enrich its content, and accelerate the research and practice of health sciences librarianship. Therefore, to avoid perpetuating or aggravating systemic biases and power structures in scholarly publishing or health sciences librarianship, we pledge to take concrete steps toward making *JMLA* a more diverse and inclusive journal.

## INTRODUCTION

Scholarly publishing is rife with systemic inequities and biases against contributors and content not representative of the majority voice [1, 2], which, in librarianship, belongs to white, middle-aged women without disabilities [3]. Recognizing that race, gender, and other personal characteristics impact who can publish, what can be published, and who gets to make the decisions, many peer-reviewed journals have made statements and taken action to increase the diversity and inclusiveness of their editorial boards, policies and processes, authors and reviewers, and published works (e.g., Flaherty [4] and *Journal of Librarianship and Scholarly Communication* editors and editorial board [5]). The *Journal of the Medical Library Association* (*JMLA*) is one of those journals.

In the summer of 2020, motivated by national conversations around Black Lives Matter, systemic racism, police violence, and health disparities laid bare by the COVID-19 pandemic, the *JMLA* team affirmed our commitment to promoting diversity and equity in health sciences librarianship and information science. We issued a call for submissions that recognize and address social injustices; speak to diversity, equity, and inclusion in our workforce and among our user populations; and share critical perspectives on health sciences librarianship as well as those on any topic within *JMLA*'s scope written by Black, Indigenous, and People of Color (BIPOC) authors [6]. Along with this call for submissions, we committed to specific actions aimed at increasing our understanding of systemic bias in scholarly publishing and creating more equitable opportunities for authors, reviewers, and editorial board members from marginalized groups. This work is led by a designated *JMLA* equity workgroup with liaisons to key caucuses, committees, and boards within the Medical Library Association (MLA).

To assess the racial diversity of recent *JMLA* authors, peer reviewers, and editorial board members and obtain baseline data for evaluating our efforts to incorporate and amplify the contributions and voices of our BIPOC colleagues, we surveyed all individuals who had served as a *JMLA* editorial board member or reviewer or had submitted a manuscript to *JMLA* between 2018 and 2020. In addition to assessing racial identity, we also took this opportunity to obtain data on gender, sexual, and disability identities and inquire about barriers to publishing in or working with *JMLA* experienced by recent authors, peer reviewers, and editorial board members.

## DEMOGRAPHIC SURVEY

We iteratively developed a survey instrument with input from all *JMLA* equity workgroup members (supplemental appendix). Our target population was individuals who served as a *JMLA* editorial board member (n=85), reviewed a manuscript submitted to *JMLA* (n=433), or submitted a manuscript to *JMLA* (including all manuscript coauthors, n=905) between 2018 and 2020. After de-duplicating the names of individuals who engaged with *JMLA* in more than 1 role, our population consisted of 1,156 unique individuals. With the assistance of MLA staff, a link to the survey in REDCap was sent to the email addresses of the 1,156 individuals on December 1, 2020. The survey closed on December 15, 2020, after email reminders were sent to nonresponders. No identifying information was collected in the survey, and respondents were assured that survey data would only be reported in aggregate.

A total of 290 respondents completed the survey; 46 indicated they had been an editorial board member (54% response rate), 162 had served as a reviewer (37% response rate), and 196 had submitted a manuscript to *JMLA* (22% response rate). For analysis and reporting purposes, we categorized authors into 2 non-mutually exclusive groups: “submitting authors” (n=196), defined as authors who submitted a manuscript regardless of the editorial decision, and “published authors” (n=167), defined as authors whose manuscripts were accepted and published. Therefore, the “published authors” group was a subset of the larger “submitting authors” group.

Closed-ended responses were analyzed with descriptive statistics and visualized using bubble plots, in which the size of the circle indicates the percentage of respondents within a certain group. For comparative purposes, we include corresponding demographic data for MLA members [7] within the bubble plots when available. However, we caution against making direct comparisons between our survey responses and MLA members, as most *JMLA* authors [8], many reviewers, and some editorial board members are not members of MLA. Open-ended responses were analyzed using informal thematic analysis.

### Racial identity

**Figure 1:**
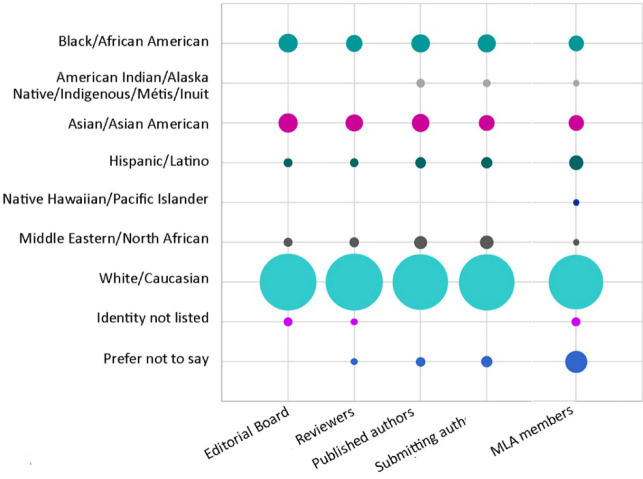
Racial identity of *Journal of the Medical Library Association (JMLA)* editorial board members, reviewers, and published and submitting authors (compared with MLA members [7])

Respondents were asked to select their racial identity from a predefined list, with multiple selections allowed. Editorial board members, reviewers, published authors, and submitting authors were predominantly White/Caucasian (78%, 80%, 77%, and 78%, respectively; Figure 1). Smaller proportions of editorial board members, reviewers, published authors, and submitting authors were Black/African American (9%, 7%, 8%, and 8%) or Asian/Asian American (9%, 7%, 8%, and 7%). Few editorial board members, reviewers, published authors, and submitting authors were Middle Eastern/North African (2%, 2%, 4%, and 5%), Hispanic/Latino (2%, 2%, 3%, and 3%), or American Indian/Alaska Native/Indigenous/Métis/Inuit (0, 0, 2%, and 2%). No respondents were Native Hawaiian/Pacific Islander (0, 0, 0, and 0). Some editorial board members, reviewers, published authors, and submitting authors indicated that they had a racial identity not listed (2%, 1%, 0, and 0) or preferred not to state their racial identity (0, 1%, 2%, and 3%). The racial identity composition of *JMLA* affiliates was largely similar to that of MLA members.

Within each group along the y-axis, the size of the bubbles indicates the percentage of respondents who selected each identity.

### Gender identity

**Figure 2:**
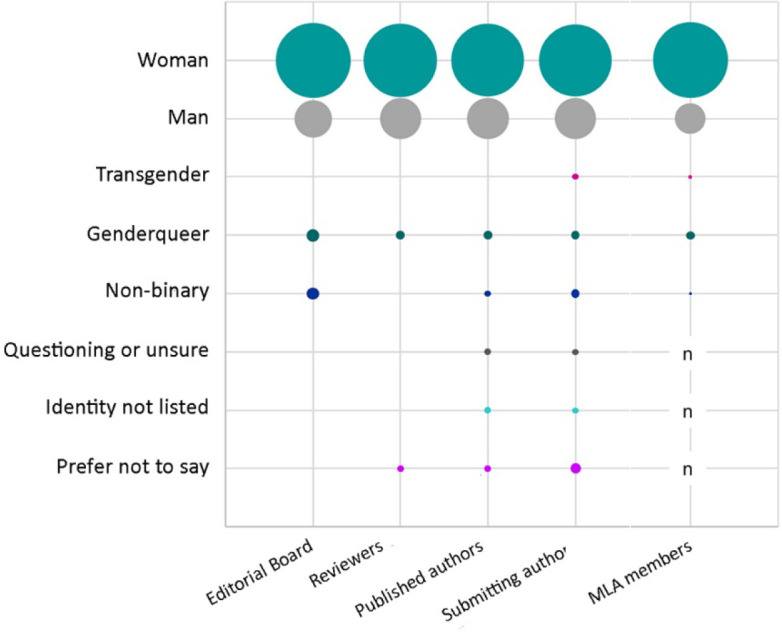
Gender identity of *JMLA* editorial board members, reviewers, and published and submitting authors (compared with MLA members [7])

Respondents were asked to select their gender identity from a predefined list, with multiple selections allowed. Editorial board members, reviewers, published authors, and submitting authors were predominantly women (78%, 75%, 74%, and 72%, respectively) (Figure 2). A smaller proportion of editorial board members, reviewers, published authors, and submitting authors were men (20%, 23%, 24%, and 23%). Few editorial board members, reviewers, published authors, and submitting authors were genderqueer (2%, 1%, 1%, and 1%), non-binary (2%, 0, 1%, and 1%), or transgender (0, 0, 0, and 1%). Some editorial board members, reviewers, published authors, and submitting authors indicated that they had a gender identity not listed (0, 0, 1%, and 1%) or preferred not to state their gender identity (0, 1%, 1%, and 2%). The gender identity composition of *JMLA* affiliates was largely similar to that of MLA members.

Within each group along the y-axis, the size of the bubbles indicates the percentage of respondents who selected each identity. n=not available.

### Sexual identity

**Figure 3:**
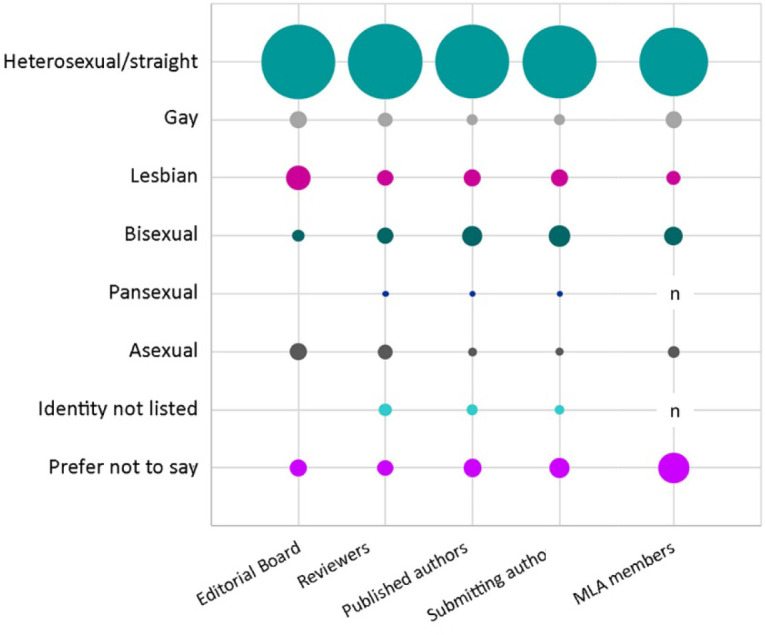
Sexual identity of *JMLA* editorial board members, reviewers, and published and submitting authors (compared with MLA members [7])

Respondents were asked to select their sexual identity from a predefined list, with multiple selections allowed. Editorial board members, reviewers, published authors, and submitting authors were predominantly heterosexual/straight (80%, 81%, 79%, and 78%, respectively) (Figure 3). Much smaller proportions of editorial board members, reviewers, published authors, and submitting authors were lesbian (9%, 4%, 4%, and 4%), bisexual (2%, 4%, 6%, and 7%), gay (4%, 3%, 2%, and 2%), asexual (4%, 3%, 1%, and 1%), or pansexual (0%, 1%, 1%, and 1%). Some editorial board members, reviewers, published authors, and submitting authors indicated that they had a sexual identity not listed (0, 2%, 2%, and 2%) or preferred not to state their sexual identity (4%, 4%, 5%, and 6%). The sexual identity composition of *JMLA* affiliates was largely similar to that of MLA members.

Within each group along the y-axis, the size of the bubbles indicates the percentage of respondents who selected each identity. n=not available.

### Disability identity

**Figure 4:**
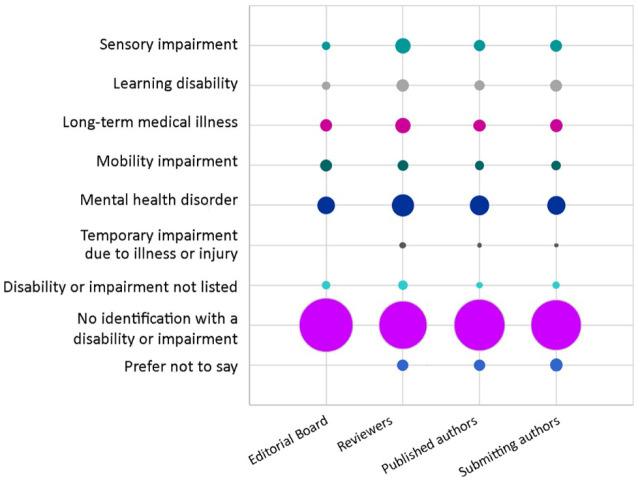
Disability identity of *JMLA* editorial board members, reviewers, and published and submitting authors

Respondents were asked to select their disability identity from a predefined list, with multiple selections allowed. Most editorial board members, reviewers, published authors, and submitting authors did not identify with a disability or impairment (80%, 65%, 74%, and 72%, respectively) (Figure 4). Of the disability identities listed, the most common was mental health disorders (9%, 14%, 11%, and 10%), followed by long-term medical illnesses such as epilepsy or cystic fibrosis (4%, 7%, 4%, and 5%); sensory impairments such as vision or hearing impairment (2%, 7%, 4%, and 4%); learning disabilities such as attention deficit hyperactivity disorder (ADHD) and dyslexia (2%, 4%, 3%, and 4%); mobility impairments (4%, 3%, 2%, and 3%); and temporary impairments due to illness or injury such as a broken ankle or surgery (0, 1%, 1%, and 1%). Some editorial board members, reviewers, published authors, and submitting authors indicated that they had a disability or impairment not listed (2%, 2%, 1%, and 2%) or preferred not to state their disability identity (0, 4%, 4%, and 5%). The disability identity composition of *JMLA* affiliates was not compared to that of MLA members due to the unavailability of detailed reporting on MLA members [7].

Within each group along the y-axis, the size of the bubbles indicates the percentage of respondents who selected each identity.

### Barriers to working with *JMLA*

The final survey item was an open-ended prompt to “Describe any barriers you have experienced to publishing in or working with *JMLA,*” to which 89 respondents provided relevant responses. Of these 89 respondents, 46 (52%) said that they encountered no barriers to publishing in or working with *JMLA*, with several individuals stating that they experienced a smooth publishing process, were treated professionally, and/or found the editors to be helpful and supportive. Ten (11%) respondents said that the only barriers they encountered were workplace or personal barriers, such as lack of research support, time, experience, or knowledge of the publishing process.

Other respondents identified barriers specific to *JMLA*. Seven (8%) respondents said that *JMLA*'s reviewers were unprofessional, too nit-picky, or untrained. Seven (8%) respondents commented on subjectivity in the reviewers’ comments or editors’ decisions, sometimes leading to decisions to decline a manuscript without sufficient rationale or based on its perceived lack of fit to *JMLA*'s scope, lack of novelty, or narrow geographical focus on non-US countries. Four respondents each said that *JMLA* did not do enough outreach to professionals in the field, particularly new librarians (5%); *JMLA* was too focused on articles describing research and/or certain topics (5%); and *JMLA*'s standards were too high (5%). Three (3%) respondents found the *JMLA* submission system to be complicated or confusing. Two (2%) respondents said that the manuscript revision process was too burdensome. One respondent each said that the author guidelines were too cumbersome (1%); making changes to article proofs was too difficult (1%); editors were not sufficiently skilled in editing manuscripts covering topics related to diversity, equity, and inclusion (1%); and the editorial board member selection process was not transparent (1%).

## CONCLUSIONS

We found that *JMLA* editorial board members, reviewers, and authors are mostly white, heterosexual women without disabilities or impairments, similar to the demographic characteristics of the MLA membership [7], academic librarianship [9], and librarianship as a whole [3]. While this finding is not surprising, it suggests that *JMLA* lacks representation and contributions from individuals who are not white, who are LGBTQ+, or who have disabilities or impairments that influence their views of, or approach to, health sciences librarianship. In other words, *JMLA* is missing out on a diversity of perspectives and life experiences that would continuously improve the journal's processes and policies, enrich its content, and accelerate the research and practice of health sciences librarianship. Therefore, we commit to developing concrete strategies and programs to become more inclusive of new voices and topics, to diversify *JMLA*'s content and contributors, and to create more equitable opportunities for *JMLA* authors, reviewers, and editorial board members.

Although most survey respondents indicated that they did not experience barriers to working with or publishing in *JMLA*, this does not prove that such barriers do not exist. Rather, it is likely that most respondents occupy positions of privilege due to their racial, gender, sexual, or disability identities and thus do not experience barriers that may stand in the way of people from marginalized groups. It is possible that individuals who encountered barriers to working with or publishing in *JMLA* were less likely to respond to our survey. Furthermore, individuals who may have encountered significant barriers preventing them from submitting to or serving in a volunteer role for *JMLA* would not have received a survey invitation. Therefore, to acquire additional feedback from a broader contingent of *JMLA* readers and potential authors, reviewers, and editorial board members, we will create an anonymous virtual suggestion box, available through the *JMLA* website, and plan to conduct another survey on barriers to publishing in or working with *JMLA* among all MLA members regardless of their role, or lack thereof, with *JMLA*.

Of the barriers to working with or publishing in *JMLA* mentioned by survey respondents, some might be expected regardless of the journal (e.g., lack of time to conduct research or write), and some could be related to *JMLA*'s premier status in the field (e.g., high standards for manuscript acceptance). However, other barriers could be products of implicit bias or structural discrimination that must be recognized and dismantled. In particular, we suspect that the respondent who stated that *JMLA* editors were not sufficiently skilled in editing manuscripts on topics related to diversity, equity, and inclusion spoke directly to our recent failure [10–12] to appropriately honor the voices or experiences of BIPOC authors seeking to publish an editorial on anti-Blackness in libraries [13]. This mistake shines a spotlight on the urgent need to (1) educate our editorial team about how our actions or inactions continue to perpetuate systemic racism and white supremacy within scholarly publishing; (2) interrogate internal *JMLA* workflows to identify and remove barriers that impede contributions from, or engagement with, BIPOC individuals; and (3) actively seek to build trust among, and form stronger partnerships with, communities of BIPOC individuals and other people who are often marginalized due to their racial, gender, sexual, or disability identities.

Charlotte Roh, scholarly communications librarian at the University of San Francisco, pointedly asks, “As librarians who are engaging more directly with scholarly publishing, we must ask ourselves: Are we perpetuating the biases and power structures of traditional scholarly publishing?” [1]. We steadfastly do not want *JMLA* to perpetuate or exaggerate systemic biases and power structures in scholarly publishing or health sciences librarianship and pledge to continue working to make *JMLA* a more diverse and inclusive journal with equitable opportunities for authors, reviewers, and editorial board members. In particular, we thank *JMLA*'s stakeholders for providing valuable guidance toward identifying and removing barriers to publishing that may stem from unconscious racism or other forms of discrimination [14], which we are actively using to scrutinize and improve our policies and procedures.

## SUPPLEMENTAL FILE

**Appendix:** *Journal of the Medical Library Association*'s editorial board, reviewer, and author survey
