# Supplementary material for: Racial, gender, sexual, and disability identities of the Journal of the Medical Library Association's editorial board, reviewers, and authors
Source: J Med Libr Assoc. 2021 Apr 1;109(2):167–73. doi: 10.5195/jmla.2021.1216 (PMC8270382; doi:10.5195/jmla.2021.1216)
Supplement: Supplementary file 1 — Appendix: Journal of the Medical Library Association's editorial board, reviewer, and author survey [file jmla-109-2-167-s01.pdf]

Journal of the Medical Library Association

Supplemental content to
J Med Libr Assoc. Apr;109(2):dx.doi.org/10.5195/jmla.2021.1216
www.jmla.mlanet.org
© Akers, Pionke, Aaronson, Chambers, Cyrus, Eldermire, Norton 2021

Racial, gender, sexual, and disability identities of the Journal of the Medical Library Association's Editorial Board, reviewers, and authors

Katherine G. Akers; JJ Pionke; Ellen M. Aaronson, AHIP; Thane Chambers; John W. Cyrus; Erin R.B. Eldermire; Melanie J. Norton

APPENDIX

***Journal of the Medical Library Association*'s editorial board, reviewer, and author survey**

Which of these roles have you held at the *Journal of the Medical Library Association (JMLA)* in the past three years? Select all that apply.

- O Editorial board member (46, 16.8%)
- O Author: my article(s) was published (167, 60.9%)
- O Author: my article(s) was submitted but not accepted (49, 17.9%)
- O Reviewer (162, 59.1%)

Which of these best describes your racial identity? Select all that apply.

- O African American/Black (18, 6.2%)
- O American Indian/Alaska Native/Indigenous/Métis/Inuit (3, 1.0%)
- O Asian American/Asian (20, 6.9%)
- O Hispanic/Latino (11, 3.8%)
- O Native Hawaiian/Pacific Islander (0)
- O Middle Eastern/North African (12, 4.1%)
- O White/Caucasian (228, 78.6%)
- O A racial identity not listed (2, 0.7%)
- O I prefer not to say (7, 2.4%)

Please specify, if desired.

Which of these best describes your gender identity? Select all that apply.

- O Woman (217, 74.8%)
- O Man (64, 22.1%)
- O Transgender (1, 0.3%)
- O Cisgender (22, 7.6%)
- O Genderqueer (2, 0.7%)
- O Nonbinary (3, 1.0%)
- O Questioning or unsure of gender identity (1, 0.3%)
- O A gender identity not listed (1, 0.3%)
- O I prefer not to say (3, 1.0%)

Please specify, if desired.

JMLA Journal of the Medical Library Association

SC
Supplemental content to
J Med Libr Assoc. Apr;109(2):dx.doi.org/10.5195/jmla.2021.1216
www.jmla.mlanet.org
© Akers, Pionke, Aaronson, Chambers, Cyrus, Eldermire, Norton 2021

How do you describe your sexual identity? Select all that apply.

- Heterosexual/straight (229, 80.4%)
- Gay (8, 2.8%)
- Lesbian (10, 3.5%)
- Bisexual (15, 5.3%)
- Pansexual (1, 0.4%)
- Asexual (6, 2.1%)
- An identity not listed (6, 2.1%)
- I prefer not to say (15, 5.3%)

Please specify, if desired.

How do you describe your disability/ability status? We are interested in this identification regardless of whether you typically request accommodation for this disability. Select all that apply.

- A sensory impairment (vision or hearing) (14, 4.9%)
- A learning disability (e.g., attention deficit hyperactivity disorder [ADHD], dyslexia) (12, 4.2%)
- A long-term medical illness (e.g., epilepsy, cystic fibrosis) (16, 5.6%)
- A mobility impairment (9, 3.2%)
- A mental health disorder (29, 10.2%)
- A temporary impairment due to illness or injury (e.g., broken ankle, surgery) (2, 0.7%)
- A disability or impairment not listed (6, 2.1 %)
- I do not identify with a disability or impairment (206, 72.5%)
- I prefer not to say (12, 4.2%)

Please specify, if desired.

Describe any barriers you have experienced to publishing in or working with *JMLA*. **Please do not share any potentially identifiable information (e.g., your name, your institution, or specific details about people involved in any experiences).**
